# The Emergence and Maintenance of Vector-Borne Diseases in the Khyber Pakhtunkhwa Province, and the Federally Administered Tribal Areas of Pakistan

**DOI:** 10.3389/fphys.2012.00250

**Published:** 2012-07-09

**Authors:** Nathan C. Nieto, Khalid Khan, Ghufran Uhllah, Mike B. Teglas

**Affiliations:** ^1^Department of Agriculture, Nutrition, and Veterinary Science, University of NevadaReno, NV, USA; ^2^Veterinary Research Institute, Khyber Pakhtunkhwa ProvincePeshawar, Pakistan

**Keywords:** *Anaplasma*, *Babesia*, Crimean-Congo hemorrhagic fever, dengue virus, Eid Islamic festival, hemoparasites, emerging and re-emerging disease

## Abstract

Human populations throughout much of the world are experiencing unprecedented changes in their relationship to the environment and their interactions with the animals with which so many humans are intimately dependent upon. These changes result not only from human induced changes in the climate, but also from population demographic changes due to wars, social unrest, behavioral changes resulting from cultural mixing, and large changes in land-use practices. Each of these social shifts can affect the maintenance and emergence of arthropod vectors disease or the pathogenic organisms themselves. A good example is the country of Pakistan, with a large rural population and developing urban economy, it also maintains a wide diversity of entomological disease vectors, including biting flies, mosquitoes, and ticks. Pathogens endemic to the region include the agents of piroplasmosis, rickettsiosis, spirochetosis, and viral hemorrhagic fevers and encephalitis. The northwestern region of the country, including the Khyber Pakhtunkhwa Province (KPK), formerly the North-West Frontier Provence (NWFP), and the Federally Administered Tribal Areas (FATA) are mountainous regions with a high degree of habitat diversity that has recently undergone a massive increase in human population density due to an immigrating refugee population from neighboring war-torn Afghanistan. Vector-borne diseases in people and livestock are common in KPK and FATA regions due to the limited use of vector control measures and access to livestock vaccines. The vast majority of people in this region live in abject poverty with >70% of the population living directly from production gained in animal husbandry. In many instances whole families live directly alongside their animal counterparts. In addition, there is little to no awareness of the threat posed by ticks and transmission of either zoonotic or veterinary pathogens. Recent emergence of Crimean–Congo hemorrhagic fever virus in rural populations, outbreaks of Dengue hemorrhagic fever have been reported in the region, and high prevalence of cattle infected and co-infected with multiple species of hemoparasites (*Theileria*, *Babesia*, *Anaplasma*). The emergence of which has followed the increased density of the rural population due to an influx of refugees from violent conflicts in Afghanistan and is exacerbated by an already impoverished society and wide diversity of potential arthropod vectors. These human outbreaks may be exacerbated by episodes of social upheaval but are also tied to the historically close association of people in the region with their livestock and subsequent zoonosis that result from spillover from co-habitation with infected domestic animals.

## Background

Recent estimates report that infectious diseases account for 26% of the global burden of morbidity and mortality to the world’s population, with lower and middle income countries suffering to a much higher degree than higher-income countries (Pinheiro et al., 2010). Vector-borne diseases specifically have been one of the leading causes of morbidity and mortality of human and animal populations since such records have been kept and in 2004 accounted for >50 million disability-adjusted life years (DALYs) (Townson et al., 2005). Mosquito-borne diseases such as malaria and dengue alone accounted for 46 million DALYs in the world. An analysis of 335 emerging infectious disease (EID) events between 1940 and 2004 demonstrated a marked increase in the number of EIDs, the majority of which were of zoonotic origin (60.3%), 71.8% from wildlife, and 22.8% of which were vector-borne (Jones et al., 2008). This study also showed that EID events are significantly associated with socioeconomic and ecological factors where regions that have lower income and in lower latitudes had a higher probability of experiencing an EID event (Jones et al., 2008). The authors generated a predictive model of EID events and generated “hot-spots” and showed that human population density correlated well to areas where EIDs are expected to result. Both Pakistan and neighboring India retained some of the highest estimates of relative risk for an EID event from all four predictive categories including vector-borne diseases, zoonosis from wildlife, zoonosis from non-wildlife, and the emergence of drug-resistant pathogens.

Vector-borne diseases at the beginning of the twentieth century often resulted in widespread epidemics affecting thousands of people throughout the world (Gubler, 1998b). In response to disease outbreaks and coinciding with a better understanding of vector-borne disease dynamics, disease control measures focusing on the elimination or reduction of vector populations were highly effective at blocking the transmission of diseases. New vaccines, insecticides, and a combination of approaches termed Integrated Pest Management (IPM) or Integrated Vector Management (IVM) have proved effective methods of reducing transmission of vector-borne pathogens (Weidhaas and Focks, 2000). Ironically, the apparent success of these efforts resulted in a reduction of control programs for malaria, yellow fever, and dengue as early as the 1970s and were largely disbanded or the diseases were no longer considered severe enough public health concerns (Gubler, 2010).

Pathogens within populations emerge for many reasons including introduction via infected humans and livestock, lack of public health resources (poverty), and changes in climate that allow for expansions of vector ranges due to changes in precipitation or temperature regimes. These interactions result not only from human induced changes in the climate, but also from population demographic changes due to wars, social unrest, behavioral changes resulting from cultural mixing, and large-scale changes in land-use practices. Changes in socio-economic status and the concentration of humans in urban environments were shown in Eastern Europe to lead to an increase in the incidence of tick-borne encephalitis (TBE) cases (Sumilo et al., 2008). The authors report that although an increase in infected wildlife or *Ixodes* ticks was not necessarily detected, other societal changes (change in land-use, reduced amount of pesticide usage, and an increase in human density) were factors responsible for bringing humans into closer contact with infected ticks (Sumilo et al., 2008). Each of these social shifts can affect the maintenance and emergence of arthropod vectors of disease or the pathogenic organisms themselves. One of the most important drivers of EID emergence will continue to be increases in human population density and the ecological shifts associated with that change.

We use Pakistan as an example of a country with a large rural population and developing urban economy that is also endemic for a wide variety of insect and tick vector species (Reisen and Boreham, 1979; Ghosh et al., 2007). Since independence in 1947, Pakistan has had a volatile history, starting with a massive influx of people from neighboring India due to religious differences (Pakistan is an Islamic Republic while India is majority Hindu). Additionally, the northwestern region of the country, including the Khyber Pakhtunkhwa Province (KPK), formerly the North-West Frontier Provence (NWFP), and the Federally Administered Tribal Areas (FATA) has recently undergone an increase in human population density due to an immigrating refugee population from neighboring war-torn Afghanistan. The migration has occurred over a period of 30 years, beginning with the Soviet invasion in 1979 and then more recently with the influx of International Security Assistance Forces (ISAF) and NATO forces into the region. This has lead to a net increase of 3–5 million refugees and their animals in and around Peshawar and the KPK (Yusuf, 1990). Peshawar is the closest large city to the Afghanistan border, directly across from the Khyber Pass, an important trade route and a porous mountainous border populated by tribal peoples from the area. The increase in population density and the poor living conditions provide two important prerequisites for the establishment of an EID or for the amplification of diseases already endemic.

The majority of the population of the KPK and FATA are Pashtun and speak the pashto language with a high diversity of dialects. Originally, their ancestors are migrants from Afghanistan, and central Asian countries that settled mostly in and around the rocky and mountainous regions of northwestern Pakistan and Afghanistan. This region stretches from the Himalayas in the north to the Sulaiman Mountain Range and Waziristan in the South. The whole province is an amalgam of lush green planes, rocky mountains, pine forests, and deserts. Agriculture is limited by the lack of arable land and irrigation water limiting the socio-economic growth for the people of province. Most of the population of the KPK and FATA depend on raising livestock for their livelihood (>70%). This province has recently suffered from natural disasters including a massive earthquake in 2010 in the northern regions of the country and a flood in the same year that affected a majority of the people and livestock living there. Agricultural land was damaged, crops were destroyed, and livestock were killed. The scenario also paved the way for EID and endemic diseases to flourish including Dengue virus (DENV), malaria, and cholera (http://www.promedmail.org/).

Vector-borne diseases in people and livestock are common in the KPK and FATA regions due to the limited use of vector control measures and access to vaccines. Human outbreaks may be exacerbated by episodes of social upheaval but are also tied to the historically close association of people in the region with their livestock and subsequent zoonosis that can result from spillover from infected domestic animals. The goals of this review are to discuss the current distribution of vector-borne diseases in KPK and FATA including the emergence and maintenance of zoonotic viruses and hemoparasites of livestock, discuss hypotheses for emergence and subsequent endemism in the region, and finally to suggest preventative measures to block transmission in humans and domestic animals.

## Dengue Virus, Dengue Fever, and Dengue Hemorrhagic Fever

Dengue virus, a member of the Flaviviridae (genus *Flavivirus*) group of viruses, is considered one of the world’s foremost EIDs (Jelinek, 2010). The virus is a single stranded, enveloped, positive strand RNA virus with four antigenically distinct serological subtypes (DEN-1–4). DENV is transmitted by mosquitoes in the genus *Aedes* (*Ae. aegypti* and *Ae. albopictus*), who preferentially feed on humans and thrive in peri-domestic environments (Kyle and Harris, 2008). In humans, the disease presents as an acute and benign febrile disease or more severely with cephalalgia, retro-ocular pain, photophobia, and muscle and joint pains commonly referred to as “bone-break fever.” In tropical Asia a severe and fatal hemorrhagic form of the disease occurs mainly in children. The current pandemic has resulted in an estimated 50–100 million cases of Dengue fever (DF) and ~250,000–500,000 cases of dengue hemorrhagic fever per year (DHF; Guzman and Kouri, 2003). The first case of DF was identified in Manila, Philippines, and since has been reported throughout Southeast Asia and then globally. In Southeast Asia the number of cases of DHF has increased from ~10,000 to more than 200,000 cases per year since it is initial discovery in the 1950s (Gibbons and Vaughn, 2002). While the exact mechanism for the emergence of DHF is still not understood, most DHF cases have been associated with either DEN-2 and DEN-3 (Gubler, 1998a), both of which have been reported circulating in Pakistan and the Indian sub-continent (Raheel et al., 2011).

The Indian sub-continent has experienced outbreaks of DF with increasing frequency since the initial discovery of the disease. While DF may have been endemic in the Indian sub-continent previously the first recorded cases of DHF occurred in Delhi in 1989 associated with DEN-2 subtype (Dar et al., 1999). Several DENV outbreaks have occurred periodically, occurring every 2–3 years following the initial event (Vijayakumar et al., 2005; Raheel et al., 2011). The first recorded outbreak of DHF in Pakistan occurred in 1994 in Karachi (Chan et al., 1995). The following year a second outbreak occurred in Balochistan in which ~1800 cases were reported. The virus may have been present before the recorded 1994 outbreak though. A prospective sero-epidemiological study of apparently healthy Pakistani residents of Karachi conducted in 1982 showed that 50–60% of the sample population had Hemagglutination Inhibition (HI) antibodies to three flaviviruses including Japanese encephalitis virus, West Nile virus, and Dengue virus (DEN-2; Hayes et al., 1982). The status of the serotypes of the outbreaks that occurred in Pakistan were not well defined, although antibodies for DEN-1 and DEN-2 subtypes were detected in patient sera from 1994 and again in 1998 (Chan et al., 1995; Akram et al., 1998). In more recent outbreaks (2005 and 2006) antibodies and PCR positive samples together detected the co-circulation of DEN-2 and DEN-3 viral subtypes (Jamil et al., 2007; Khan et al., 2008). In a 2008 outbreak in Punjab, DEN-2–4 subtypes were detected in patient sera and was associated with DHF cases (Humayoun et al., 2010).

In the Fall of 2011, DENV outbreaks in Pakistan surged once again causing over 15,000 cases in Lahore alone and >200 in Peshawar and the KPK (Rai, 2011). The magnitude of that year’s epidemic followed a year where most of the country was affected by massive flooding creating unprecedented mosquito habitat. The vector of DENV, *Ae. aegypti* and *Ae. albopictus*, are common human bitters and survive well in peri-domestic environments including shipping containers, human detritus, and potted house plants. Little information is available for the distribution of DENV vectors in the FATA, although one study identified *Ae. albopictus* and four other *Aedes* spp. mosquitoes from the KPK, formerly NWFP (Suleman and Shafqat, 1993). In most endemic areas, DENV outbreaks are associated with the seasonally wet and warm periods and do not typically occur during cold dry periods. However, evidence suggests that transovarial transmission of the virus can occur in both *Ae. aegypti* and *Ae. albopictus* allowing for mosquitoes eggs that survive the cold dry periods to serve as the following years infected cohort (Gunther et al., 2007). This is however dependent on a permissive climate. Both mosquito species, once infected remain infected for life (Kyle and Harris, 2008). However, in tropical areas the longest-lived *Aedes* mosquito lasted 174 days and the average life-span of a single adult mosquito is approximately 2 weeks (Kyle and Harris, 2008) making overwintering of infected adult mosquitoes unlikely. In the KPK we hypothesize that outbreaks in the large cities of the tropical areas of the country (Lahore and Karachi) spillover into more rural populations via human and animal travel along established trade routes into the KPK. DENV can then infect local *Ae. albopictus* and transient populations of *Ae. aegypti* leading to outbreaks in humans. Now that both serotypes occur in the region we will most likely observe an increase in the amount of DHF cases.

Outside of the KPK, multiple control strategies have been employed in Pakistan including pesticide treatment, larval mosquito habitat removal, and community education campaigns. While pesticide treatment is commonly used to combat mosquito populations in endemic regions, current evidence suggests resistance of *Ae. albopictus* to a number of agrochemicals in Pakistan (Khan et al., 2011). Although larval habitat removal and public health education have served as valuable tools to combat the disease in other countries (Itrat et al., 2008), this may be an unrealistic in the KPK and FATA where there is limited access for vector control agencies. Itrat et al. (2008) identified that education programs disseminated via television may be a more effective method of public education regarding larval habitat control than earlier efforts using newspapers due to a high illiteracy rate. People in the area are willing to participate in eradication efforts but unfortunately education programs are effective at educating only a limited proportion of society and there for less effective. This is especially true in KPK and FATA where most individuals are largely agrarian, migratory, and illiterate.

## Crimean–Congo Hemorrhagic Fever Virus

Crimean–Congo hemorrhagic fever virus (CCHFV, family Bunyaviridae, genus *Nairovirus*) is a tick-borne RNA virus and causes severe illness throughout Africa, Asia, Southeast Europe, and the Middle East (Whitehouse, 2004). It was first described in Crimea in the Soviet Union in 1944 and shortly thereafter an identical virus was described in the African Congo (Casals, 1969). The virus mainly affects mononuclear phagocytes, endothelial cells, and hepatocytes, causing severe hemorrhagic fever with case fatality rates ranging from 3 to 30% (Ergonul, 2006). Additionally, case fatality estimates following nosocomial infections in people who provide primary care are especially dangerous, and may reach up to 80% in some areas (Swanepoel et al., 1989; Fisher-Hoch et al., 1992; Ergonul, 2006). People become infected with the virus by exposure to blood and body fluids of infected livestock, by nosocomial infections, or by the bite of an infected tick (Burney et al., 1980; Whitehouse, 2004). The primary tick vectors of the virus are ticks in the genus *Hyalomma* and the distribution of the disease closely resembles that of the tick vector (Hoogstraal, 1979). It is hypothesized that livestock, small mammals (hares), and migrating birds serve as the reservoir hosts for the disease and display asymptomatic infections even when they have high viremic loads circulating in the blood (Hoogstraal, 1979; Swanepoel et al., 1983). *Hyalomma* is a generalist feeder and may readily feed on infected livestock or birds during one blood meal and then on humans during a subsequent feeding. The virus can be maintained via transstadial and limited transovarial transmission within the tick, and therefore once established in a region may remain, circulate, and become endemic within tick populations (Gonzalez et al., 1992).

Although the distribution of CCHFV is worldwide, with the exception of the Americas, there has been a recent emergence of the virus in areas where infection has not historically been common, including Greece (2008), Georgia (2009), Turkey (2002), Iran (2003), and Pakistan (2005; Saleem et al., 2009; Carroll et al., 2010; Mild et al., 2010). Pakistan has had 14 CCHFV outbreaks to date and of those, half have occurred since the year 2000 (Rai et al., 2008). In addition to an increased outbreak frequency there also appears to be an increase in the virulence of clinical disease with mortality rates since 2002 reaching as high as 75%, as opposed to earlier reports of 5–30%. Nosocomial transmission reportedly has a much higher mortality rate and many of the severe outbreaks in Pakistan have included infection amongst health care providers (Rai et al., 2008). Causes of emergence of CCHFV are varied and include changes in vector and reservoir host distribution due to changing climactic conditions, reservoir host migration patterns, and especially the movement of people and their animals from endemic to non-endemic regions (Mild et al., 2010).

In Pakistan, CCHFV outbreaks have occurred sporadically since it is initial discovery in 1976 in Rawalpindi (Burney et al., 1980). While the virus is considered enzootic in Balochistan, other areas in the north of the country including the cities of Abbottabad and Peshawar (KPK) have recently experienced deadly outbreaks (Saleem et al., 2009). CCHFV outbreaks typically occur following the migrations of nomadic people and livestock to district centers where they bring animals to sell and slaughter. This occurs following times of social upheaval (i.e., immigration from neighboring Afghanistan and following the 2005 earthquake) as well as during the culmination of the Islamic festival of Eid-ul-Azha (Smego et al., 2004; Rai et al., 2008; Saleem et al., 2009). In both cases livestock and people leave the rural country-side to travel to the large city centers of each district where animals are sold and slaughtered. For example, in the village of Bannu, the average number of sheep, goats, and cattle within the city can increase 100-fold during the few days preceding the Eid-ul-Azha festival. Abattoir workers are commonly infected during slaughter of viremic animals and during the Eid-ul-Azha, animal slaughter in individual homes can serve as a source of transmission to those who may not normally be at increased risk of infection (Rai et al., 2008). In addition, due to the porous nature of the Pakistan-Afghan border livestock and people from neighboring Afghanistan also bring their animals to market in Pakistan or graze their animals in the Pakistani country-side. This disease has been report in Afghanistan where in 2009 a US soldier acquired a CCHFV infection (Olschlager et al., 2011). The effect of increased movements of people and livestock due to the war in Afghanistan may have resulted in the establishment of infected ticks and/or livestock to a previously naïve region, namely the KPK and FATA Provinces in northwestern Pakistan.

The risk of CCHFV infection is directly related to the density of vectors available for human contact and the prevalence of CCHFV in the vector populations (Hoogstraal, 1979). The prevalence in the vector population is indirectly related to the prevalence of CCHFV in wild animal reservoir populations and the density of livestock that can maintain the vector-pathogen-host cycle. Tick populations are regulated by the availability of hosts for feeding and by individual tick survival both of which vary depending on climactic conditions and differential habitat quality (Sonenshine, 1992). Outbreaks of CCHFV in Pakistan appear to occur following the migration of rural people and their animals into the cities during religious celebrations or strife exposing urban populations to either exposure to viremic fluids from slaughtered animals or through the bites of infected ticks transported by infested livestock. CCHFV human cases primarily occur during the months of October and November (Jamil et al., 2005), which often corresponds to the season where the Eid-ul-Azha celebration and can result in large numbers of animals brought to the cities from rural areas for sacrifice (Rai et al., 2008). In both the KPK and FATA the timing of DHF outbreaks occur most frequently during August, September, and October (Riaz et al., 2009). The outbreak of DHF is associated with an increase in mosquito breeding habitat following the monsoon rains of these months (Riaz et al., 2009), which often ends just prior to the Eid celebration. The clinical presentation of both diseases is also similar. Both diseases are reported most commonly in young males, presenting with fever, vomiting, and diarrhea (Jamil et al., 2005; Butt et al., 2008). CCHFV may also lead to bleeding from body orifices (~50% cases) while DHF results in mucosal bleeding and a rash in 80% of patients although early on in the infection may appear the same (Butt et al., 2008; Riaz et al., 2009; Saleem et al., 2009). These subtle differences in presentation may lead to dangerous consequences for primary care givers as CCHFV may be transmitted directly. Diagnosis of either virus infection can be quickly accomplished with molecular methods however there is limited availability of this type of diagnosis in Pakistan, especially in the FATA and KPK regions (Smego et al., 2004).

## Emerging Vector-Borne Hemoparasites

Hemoparasitic diseases caused by vector-borne parasites (protozoans and bacteria) constitutes a disease entity of considerable public and veterinary health, and economic importance in tropical and subtropical regions (Colwell et al., 2011). While many are of obvious importance in Pakistan and globally (malaria, leishmania, and borreliosis), a large number of novel pathogens are being identified in animal populations that have not been reported previously due to improved diagnostic analysis made possible by molecular genetic tools. In fact, hemoparasitic pathogens serve as a limiting factor in maintaining exotic cross-bred cattle, local cattle breeds, buffaloes, and small ruminants in many subsistence agricultural communities. Economic losses result both directly and indirectly from the morbidity and mortality caused by the high incidence of these diseases and from the animals serving as a reservoir of zoonotic vector-borne agents (Irwin and Jefferies, 2004; Otranto et al., 2009). In Pakistan, the increased immigration of people resulted in a concomitant increase in the number of livestock. There for accidental transportation of pathogen vectors on livestock and infected animals could account for the spread of zoonotic pathogens and their establishment in an area in which it had not been present previously. Additionally, the importation of naive animals to a region where an endemic disease occurs could result in the exposure of new hosts and amplification of a pathogen. This scenario has been realized in piroplasmosis epidemics previously (Caracappa, 1999; Hofle et al., 2004) although little information is available in Pakistan.

In the KPK and FATA, livestock can be infected by multiple blood-borne parasites including those that cause piroplasmosis (*Babesia* and *Theileria* spp) and Anaplasmosis (caused by *Anaplasma marginale, A. central*, and *A. phagocytophilum* in sheep and goats), all of which can cause severe disease in animals and potential production losses (Khan, 1991). *Babesia* spp. and *A. phagocytophilum* are recognized as human zoonotic pathogens in the region and diagnosis in the human cases can be subtle and difficult. Heamoparasitic disease occurs sporadically and cases are identified throughout the year (Buriro et al., 1994; Khan et al., 2004). The prevalence of *Babesia* and *Anaplasma* in the people of KPK and FATA is unknown, but given the widespread prevalence in animals humans are surely affected.

Intraerythrocytic parasites in the genus *Babesia* infect domestic animals and causes severe anemia and hemoglobinuria. The pathology of this disease is due to the dividing parasites within erythrocytes that produce rapid destruction of the cells and accompanying hemoglobinemia, hemoglobinuria, fever, and lethargy (Beattie et al., 2002). Acute cases lead to death in 20% of untreated infected animals, parasitemia may involve between 0.2 and 45% of erythrocytes (Mahoney, 1977). *Babesia* spp. are transmitted by the bite of a tick vector, namely due to hard tick vectors (Ixodidea) and is maintained transovarially (Ruebush et al., 1981). The disease babesiosis, appears particularly severe in naïve animals when they are introduced into an endemic area, and is a considerable constraint on livestock development in many parts of the world. In Pakistan, *B. bovis, B. bijemina*, and *B. ovis* are most commonly identified infecting livestock (Khan, 1991). In North America and Europe disease in humans has been caused by *B. microti* and *B. divergens* respectively (Hunfeld et al., 2008). The reservoir hosts include wild rodents, wild ungulates, and Bovids (Mahoney, 1977). Babesiosis has increasingly been identified in North America and Europe as a zoonotic disease due to an increased ability to diagnose infection with molecular methods. Because of the subtle effects of anemia, disease in humans can be misdiagnosed, but with the emergence of immunosuppressive pathogens like HIV, infection with *Babesia* spp. may become more common (Harrus and Baneth, 2005; Rai et al., 2007).

Anaplasmosis caused by the rickettsial bacteria, *A. marginale* and *A. centrale* in large ungulates and *A. phagocytophilum* in sheep and goats, has also been identified in livestock in Pakistan. *Anaplasma* spp. belong to the family Rickettsiaceae bacteria that are obligately intracellular and also difficult to diagnose. In fact, 53% of the EID events that have occurred over the past 50 years were caused by *Rickettsia* (Jones et al., 2008). The parasites are cosmopolitan in nature, and the disease often co-exits with babeseosis due to a shared Ixodid vector (*Ixodes persulcatus* throughout Asia), but single species infections predominate. This disease is characterized by acute anemia, fever, jaundice and the degeneration of the internal organs. Naturally buffaloes, bison, various antelope species, deer, elk, camels, goats, and sheep are all susceptible to anaplasmosis while *A. phagocytophilum* usually has a natural reservoir in wild rodents (Foley et al., 2008). The emergence has been associated with an increase in the presence of wild rodent hosts in regions where the tick vector is already abundant (Chen et al., 1994). Again while the pathogen does exist in sheep no evidence of infection has been identified, although as mentioned earlier, with the emergence of HIV we expect the emergence of these other tick-borne pathogens (Harrus and Baneth, 2005; Rai et al., 2007).

## Conclusions

Control of vector-borne disease in regions such as the KPK and FATA is made more difficult due to an already frail health infrastructure and because these diseases manifest with a wide variety of symptoms making misdiagnosis or inappropriate clinical treatments more likely. At the same time, a diversity of pathogens exist in a region that has suffered from political instability, refugee immigration, and poverty for decades which often results in limited access to health care and, presentation of patients to medical facilities late in the course of disease resulting in higher mortality rates (Rai and Khan, 2007). The majority of the human population in the KPK and FATA live in poverty with >70% of the population living directly from production gained in animal husbandry. In many instances whole families live directly alongside their animal counterparts. The increase in human density has lead directly to an increase in livestock density in the area. These conditions allow for the establishment of pathogens and potentially lead to increased transmission risk of zoonotic and vector-borne diseases through this close association between people and their livestock.

Residents of the KPK and FATA have limited access to vaccinations and acaracides due to the relatively high cost of these preventatives and because of the difficulties associated with the transportation and distribution of these products when available. Control efforts are hampered by the lack of public awareness in local residents to the threat posed by tick bites and the transmission of zoonotic or veterinary pathogens. This is compounded with the region’s endemicity for a variety of pathogenic agents including those that cause piroplasmosis, rickettsiosis, viral hemorrhagic fevers, and encephalitis. Although few resources are currently available to stop transmission allowing many problems to flourish, we suggest a combination of preventative and surveillance measures can be used to decrease the risk to human and animal residents of the region. Beginning with vector control efforts based on protecting livestock from acquiring infection via the bites of infected ticks. Veterinarian and public health agencies can use relatively inexpensive methods to eradicate ticks from domestic livestock through the use of topical acaricides and insecticidal drenches or dip tanks. Protecting livestock from tick bites can also serve to lessen the number of infected ticks potentially available to transmit disease to human populations. Surveillance programs tied closely with communication between medical doctors, veterinarians, and public health officials are necessary in order to develop predictive models of disease occurrence that enable public health agencies to focus their limited resources during control efforts. For many of these diseases public education at local markets and in local mosques in regards to proper biosafety precautions would be a valuable addition to most vector-borne disease control programs.

The rapid evolution of molecular genetic technologies has enabled diagnostic laboratories outside of developed countries to provide quick and accurate testing of clinical samples but their use in surveillance and disease prevention programs has not kept pace (Harrus and Baneth, 2005). These technologies can and should be applied to address the health and economic needs of the people in this region. Simple preventative measures such as these may not be sufficient to eradicate these diseases themselves but may help to alleviate human suffering in the endemic regions and possibly slow future transmission into regions of the country that are not currently affected.

## Conflict of Interest Statement

The authors declare that the research was conducted in the absence of any commercial or financial relationships that could be construed as a potential conflict of interest.
